# Delayed anti–vascular endothelial growth factor therapy in patients with type 2 diabetes mellitus and branch retinal vein occlusion–associated macular edema negatively affects visual outcomes

**DOI:** 10.3389/fendo.2026.1710295

**Published:** 2026-03-10

**Authors:** Yue Deng, Yanyu Liu, Xia Li, Liting Song, Xiaoling Zhang

**Affiliations:** Ophthalmology Center, Xinjiang 474 Hospital, Urumqi, China

**Keywords:** anti–vascular endothelial growth factor, branch retinal vein occlusion, delayed treatment, macular edema, ranibizumab, visual improvement

## Abstract

**Objective:**

To investigate the effect of delayed treatment on six-month visual outcomes in patients with type 2 diabetes mellitus (T2DM) complicated by branch retinal vein occlusion-associated macular edema (BRVO-ME).

**Methods:**

This single-center retrospective cohort study included 148 patients diagnosed with BRVO-ME and receiving their first intravitreal ranibizumab injection between January 2024 and May 2025. Patients were stratified according to the interval from diagnosis to initial anti-vascular endothelial growth factor (VEGF) injection into early treatment (≤7 days, n = 67), intermediate treatment (8–14 days, n = 52), and delayed treatment (>14 days, n = 29) groups. Baseline demographic, systemic, and ophthalmic parameters were collected. Best-corrected visual acuity (BCVA) and central macular thickness (CMT) (μm) were recorded at 1, 3, and 6 months post-injection. Multivariable linear regression was used to identify independent factors affecting six-month BCVA improvement.

**Results:**

All groups exhibited BCVA improvement and CMT reduction at six months, with the early treatment group showing the greatest improvement. Multivariable regression identified delayed treatment as the only independent factor associated with six-month BCVA improvement (β = −0.008, 95% *CI*: −0.014 to −0.002, *P* = 0.010). ROC analysis showed an AUC of 0.823 (95% *CI*: 0.751–0.884, *P* < 0.001) for delayed treatment predicting insufficient six-month visual improvement, with a sensitivity of 89.4% and specificity of 61.4% using a 14-day cutoff.

**Conclusions:**

Initiation of anti-VEGF therapy within seven days of diagnosis is associated with greater six-month visual improvement in patients with BRVO-ME and T2DM. Delayed treatment may contribute to suboptimal visual recovery.

## 1 Introduction

Branch retinal vein occlusion (BRVO) is the second most common retinal vascular disorder after diabetic retinopathy, with a global annual incidence of approximately 0.4%–1.1%, and occurs more frequently in the elderly and in patients with concomitant metabolic diseases (1). The pathogenesis of BRVO is mainly related to local venous outflow obstruction, increased retinal capillary permeability, and secondary macular edema (ME), with ME being the leading cause of visual decline in BRVO patients (2). In patients with type 2 diabetes mellitus (T2DM), the presence of microvascular changes and a hypercoagulable state further increases the risk of BRVO (3) and may exacerbate macular exudation and edema, resulting in more severe visual impairment.

In recent years, intravitreal injection of anti–vascular endothelial growth factor (VEGF) agents has become the standard treatment for BRVO-associated ME. Numerous clinical studies have demonstrated that anti-VEGF therapy significantly improves best-corrected visual acuity (BCVA) and reduces central macular thickness (CMT) (4–6). Among commonly used agents, ranibizumab has shown favorable efficacy and safety. However, these studies were mostly conducted under the premise of early initiation of therapy after diagnosis, and the impact of delayed anti-VEGF treatment on visual outcomes remains inadequately explored.

In real-world clinical practice, there is often a delay from diagnosis to the first anti-VEGF injection due to factors such as long referral times, financial burden, poor patient adherence, and uneven distribution of medical resources (7). Previous studies have suggested that in retinal vein occlusion and other retinal vascular diseases (e.g., neovascular age-related macular degeneration, diabetic macular edema), delayed treatment may expose the retina to prolonged high levels of VEGF and inflammatory cytokines, leading to irreversible photoreceptor damage (8). One study on retinal disorders reported that delaying the first anti-VEGF injection for ≥7 days was significantly associated with increased macular structural damage and limited visual recovery (9).

However, in the specific population of T2DM patients with BRVO-ME, the effects of delayed treatment may be more complex. Diabetic patients inherently exhibit retinal capillary basement membrane thickening, endothelial dysfunction, and heightened inflammatory responses, which not only worsen macular edema following venous occlusion but may also reduce the responsiveness of retinal tissue to therapy (10). Therefore, the same delay may result in more pronounced adverse visual outcomes in diabetic BRVO patients compared with non-diabetic BRVO patients.

At present, systematic studies on the impact of delayed anti-VEGF therapy on visual outcomes in diabetic patients with BRVO are limited, particularly those directly comparing different delay intervals. Furthermore, it remains unclear whether delayed treatment increases the recurrence rate of macular edema, affects the total number of injections required, or is modified by different types of anti-VEGF agents. Against this background, the present study aims to investigate the influence of anti-VEGF treatment timing on visual prognosis and analyze potential associated risk factors, providing evidence to optimize clinical treatment strategies.

## 2 Materials and methods

### 2.1 Study participants

This single-center retrospective cohort study included 148 patients (148 eyes) who were first diagnosed with T2DM complicated by BRVO-ME and received anti–VEGF therapy at the outpatient or inpatient ophthalmology department of our hospital between January 2024 and May 2025.

Sample size calculation: The primary endpoint of this study was six-month improvement in BCVA (ΔLogMAR) among different treatment delay groups. Based on previous literature (11), the expected mean ΔLogMAR difference among the three groups was 0.1, with a standard deviation of 0.15. One-way analysis of variance (ANOVA) was used for group comparisons, with α = 0.05 and statistical power = 0.90. Sample size estimation was performed using G*Power 3.1 software, resulting in a minimum of 40 patients per group and a total of 120 patients. Considering potential 10%–15% loss to follow-up and missing data in retrospective studies, the final sample size was set at no less than 135 patients. The actual study included 148 patients, meeting the requirements for statistical analysis.

The study protocol was approved by our hospital’s Ethics Committee, and the study was conducted in accordance with the ethical principles of the Declaration of Helsinki. Given the retrospective design, all data were extracted from existing clinical records and anonymized to protect patient privacy, and the requirement for written informed consent was waived.

Inclusion criteria were: (1) age ≥18 years; (2) confirmed diagnosis of T2DM according to the American Diabetes Association (ADA) standards (12); (3) confirmed BRVO with macular edema, with optical coherence tomography (OCT) showing CMT ≥300 μm and macular fluid accumulation (intraretinal fluid, IRF, or subretinal fluid, SRF) or disruption of the foveal inner retinal layers (disorganization of the retinal inner layers, DRIL) (13); (4) first-time anti-VEGF treatment and a minimum follow-up of six months.

Exclusion criteria included: (1) coexisting ocular diseases such as stage III or higher diabetic retinopathy, retinal tear, or neovascular age-related macular degeneration; (2) prior ocular treatment including intravitreal injection, laser, or vitrectomy; (3) incomplete records or insufficient follow-up; (4) media opacity interfering with OCT imaging; (5) allergy to anti-VEGF agents; (6) contraindications to ocular injection; (7) presence of malignancy, autoimmune disease, or severe infection; (8) cognitive or psychiatric disorders preventing cooperation with treatment; (9) macular edema secondary to uveitis, optic neuropathy, or other causes.

### 2.2 Grouping

Based on literature indicating that treatment delays exceeding seven days may significantly affect retinal structure and therapeutic response (9), and in accordance with clinically observed delay ranges, patients were divided into three groups according to the interval from diagnosis to first anti-VEGF injection:

Early treatment group: (1) Early treatment-within ≤7 days of diagnosis; (2) Intermediate treatment group-treatment 8–14 days after diagnosis; and (3) Delayed treatment group-treatment >14 days after diagnosis.

In this study, “time of diagnosis” was defined as the date on which BRVO-associated macular edema was first confirmed at our center based on fundus examination and OCT findings. The interval from diagnosis to first injection therefore reflects the actual waiting time within the medical system. The onset of symptoms was not used as a grouping variable because it is subjective and often unreliable in retrospective records.

### 2.3 Data collection

Data were extracted from the electronic medical record system, including: (1) Baseline demographic and systemic data: sex, age, duration of diabetes, glycated hemoglobin (HbA1c), systolic and diastolic blood pressure, lipid profile, and smoking history. (2) Baseline ophthalmic assessment including BCVA (assessed using the Early Treatment Diabetic Retinopathy Study [ETDRS] chart and converted to logarithm of the minimum angle of resolution [LogMAR] units) and CMT (measured by optical coherence tomography [OCT] in μm). (3) Follow-up indicators: BCVA and CMT at 1, 3, and 6 months after treatment, as well as the total number of anti-VEGF injections. Patients were followed up at 1, 3, and 6 months after the first intravitreal anti-VEGF injection. All follow-up time points were calculated from the date of the first injection, rather than from the time of diagnosis. (4) Macular edema recurrence (defined as CMT increase ≥50 μm accompanied by a visual acuity decline ≥0.1 LogMAR).

### 2.4 Treatment protocol

All included patients received intravitreal ranibizumab (Lucentis, Novartis, Basel, Switzerland) injections. The initial regimen followed a “3+PRN” protocol: all patients received three consecutive monthly injections of ranibizumab (0.5 mg/0.05 mL per injection) following diagnosis, followed by a pro re nata (PRN, as-needed) phase. PRN injections were determined based on OCT findings and changes in BCVA. Indications for repeat injection included: (1) CMT increase ≥50 μm compared with the previous visit; (2) LogMAR BCVA decrease ≥0.1 with persistent or recurrent macular edema on OC; (3) Evidence of active retinal leakage.

All injections were performed under sterile conditions in an operating room. Preoperatively, the ocular surface and conjunctival sac were disinfected with povidone-iodine, and 0.5% topical lidocaine was applied. A 30-gauge needle was used for pars plana intravitreal injection 3.5–4.0 mm posterior to the limbus. Post-injection, levofloxacin eye drops were administered for 3–5 days to prevent infection. Patients underwent routine follow-up at 1, 3, and 6 months, including OCT and BCVA assessment. If adverse events occurred (e.g., elevated intraocular pressure, vitreous opacity, anterior chamber inflammation), treatment was paused or discontinued and individualized management was provided.

### 2.5 Statistical analysis

All statistical analyses were performed using SPSS 26.0 software (IBM Corp., Armonk, NY, USA). Continuous variables were tested for normality; normally distributed data are presented as mean ± standard deviation ( $\bar{x}$ ± s), and non-normal data as median (interquartile range). Between-group comparisons were performed using one-way ANOVA or Kruskal–Wallis H test. Repeated measures analysis of variance was used for multi-timepoint data. Categorical variables were compared using chi-square test or Fisher’s exact test and expressed as n (%). Correlations were analyzed using Pearson’s method. Multivariable linear regression was used to evaluate independent effects of main variables on outcome measures. Receiver operating characteristic (ROC) curves were constructed to assess predictive performance. All tests were two-sided, and P < 0.05 was considered statistically significant.

## 3 Results

### 3.1 Baseline characteristics

According to treatment timing, 67 patients were included in the early treatment group, 52 in the intermediate group, and 29 in the delayed group. There were no significant differences among the three groups in age, diabetes duration, HbA1c, systolic blood pressure (SBP), diastolic blood pressure (DBP), lipid levels, smoking history, or sex distribution (*p* > 0.05) (**Table 1**). In the delayed-treatment group, the median interval from diagnosis to first injection was 36 days (interquartile range, 33–40 days), with a maximum delay of 46 days.

**Table 1:** Comparison of baseline characteristics.

Variable	Early - stage group (n=67)	Mid - stage group (n=52)	Delayed group (n=29)	F/χ²	P_value
Age	65.60 ± 5.10	64.45 ± 5.98	66.56 ± 7.23	1.274	0.283
Diabetes duration	10.36 ± 4.43	10.18 ± 5.44	10.21 ± 5.30	0.020	0.980
HbA1c	8.03 ± 1.07	8.06 ± 1.02	8.11 ± 1.12	0.059	0.943
SBP	135.54 ± 12.89	135.08 ± 10.62	138.10 ± 10.13	0.684	0.506
DBP	80.42 ± 8.16	81.21 ± 8.35	79.55 ± 7.23	0.406	0.667
Lipid	3.09 ± 0.55	3.07 ± 0.59	3.04 ± 0.55	0.082	0.922
Baseline_bcva	0.62 ± 0.18	0.60 ± 0.16	0.67 ± 0.20	1.654	0.195
Baseline_cmt	448.54 ± 69.20	468.23 ± 63.47	473.72 ± 75.20	1.895	0.154
Sex (Female/Male)	25 (37.3%)/42 (62.7%)	10 (19.2%)/42 (80.8%)	9 (31.0%)/20 (69.0%)	4.612	0.100
Smoking history	21 (31.3%)	20 (38.5%)	9 (31.0%)	0.785	0.675

### 3.2 Comparison of visual outcomes among groups

**Figure 1:**
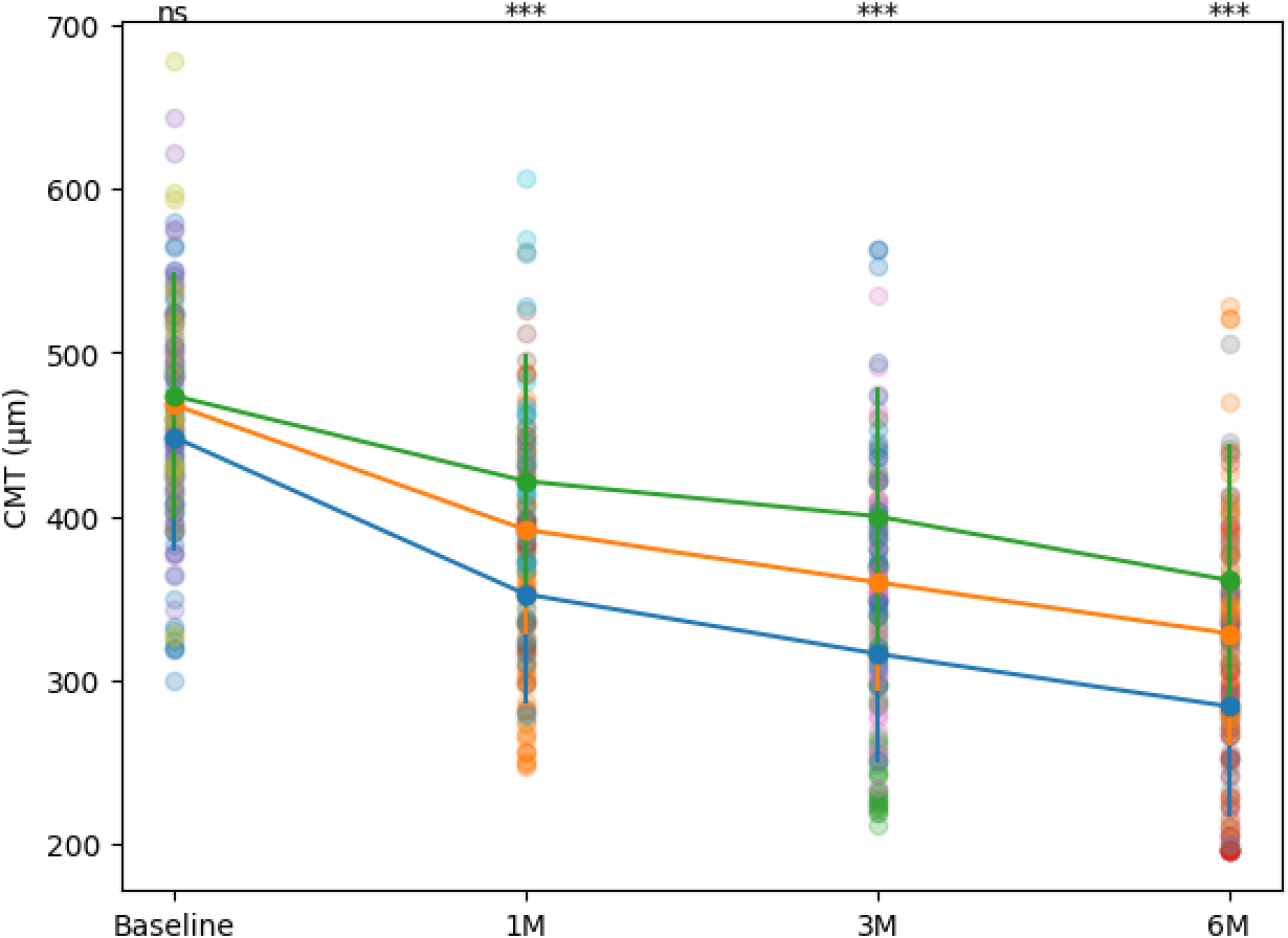
BCVA over time in the three groups. Data are presented as mean ± SD with individual patient values overlaid. 1M, 3M, and 6M represent 1 month, 3 months, and 6 months after treatment, respectively; Statistical annotations indicate pairwise comparisons between the early- and delayed-treatment groups (ns, not significant; ***P < 0.001).

During follow-up at 1, 3, and 6 months, all three groups showed a progressive improvement in BCVA (LogMAR) (**Figure 1**). The early treatment group exhibited greater visual improvement at all time points compared with the intermediate and delayed groups. Pairwise comparisons showed no significant difference in BCVA between the early- and mid-stage groups at any time point (all P > 0.05). At 6 months, the difference in BCVA improvement (ΔLogMAR) among the three groups was statistically significant (*P* < 0.001) (**Figure 1**; **Table 2**). Additionally, 73.1% of patients in the early treatment group achieved ≥15 ETDRS letter improvement (approximately ΔLogMAR ≥0.3), which was significantly higher than the intermediate group (53.8%) and delayed group (34.5%) (*P* < 0.05) (**Table 3**).

**Table 2:** Comparison of visual outcomes (BCVA, LogMAR) among groups.

Timepoint	Early - stage group (n=67)	Mid - stage group (n=52)	Delayed group (n=29)	F/χ²	P_value
Baseline	0.62 ± 0.18	0.60 ± 0.16	0.67 ± 0.20	1.654	0.195
1 month	0.39 ± 0.18	0.44 ± 0.18	0.57 ± 0.21	9.282	<0.001
3 months	0.32 ± 0.19	0.36 ± 0.20	0.51 ± 0.24	9.473	<0.001
6 months	0.27 ± 0.20	0.31 ± 0.21	0.46 ± 0.22	8.991	<0.001
≥15 ETDRS letter improvement n(%)	49 (73.1%)	28 (53.8%)	10 (34.5%)	20.580	<0.001

**Table 3:** Comparison of CMT changes among groups.

Timepoint	Early - stage group (n=67)	Mid - stage group (n=52)	Delayed group (n=29)	F	P_value
Baseline	448.54 ± 69.20	468.23 ± 63.47	473.72 ± 75.20	1.895	0.154
1 month	352.67 ± 66.22	392.02 ± 63.59	421.62 ± 76.97	11.812	<0.001
3 months	316.27 ± 66.16	359.85 ± 66.63	400.10 ± 78.51	16.176	<0.001
6 months	284.18 ± 67.22	328.65 ± 68.33	361.14 ± 83.05	13.425	<0.001
Mean CMT reduction	164.36 ± 33.90	139.58 ± 40.30	112.59 ± 38.03	20.773	<0.001

### 3.3 Comparison of CMT changes among groups

**Figure 2:**
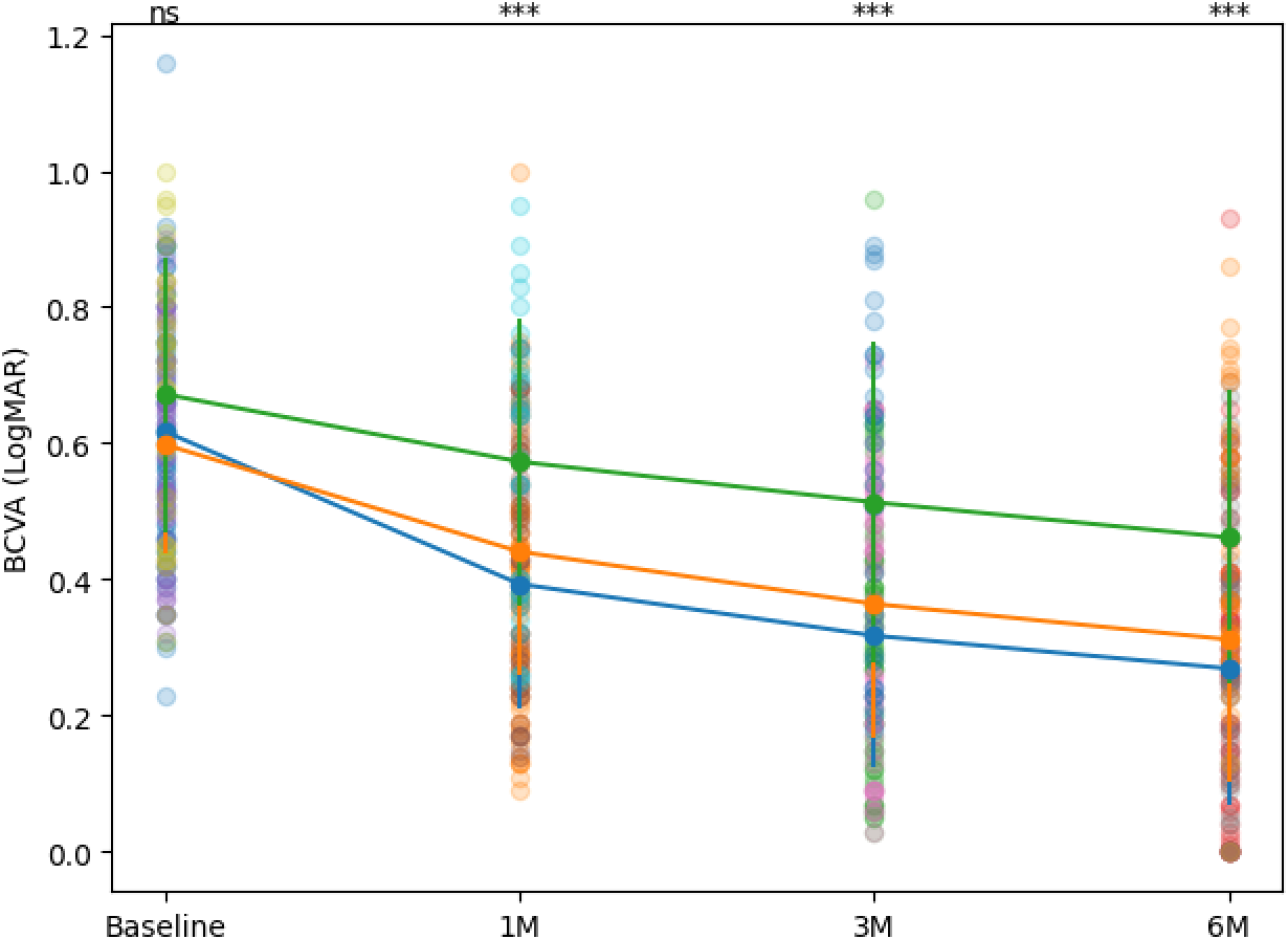
CMT over time in the three groups. Data are presented as mean ± SD with individual patient values overlaid. 1M, 3M, and 6M represent 1 month, 3 months, and 6 months after treatment, respectively; Statistical annotations indicate pairwise comparisons between the early- and delayed-treatment groups (ns, not significant; ***P < 0.001).

CMT decreased significantly after treatment in all groups, showing a time-dependent reduction (**Figure 2**). The early-treatment group showed a greater magnitude of CMT reduction than the other two groups at each follow-up. Pairwise comparisons revealed that the early-treatment group exhibited significantly greater CMT reduction than the mid-stage group at 1, 3, and 6 months (all P < 0.01), whereas no difference was observed at baseline. At 6 months, the mean CMT reduction was −164.3 ± 35.1 μm in the early group, −139.2 ± 42.8 μm in the intermediate group, and −112.5 ± 46.7 μm in the delayed group, with significant intergroup differences (*P* < 0.001).

### 3.4 Comparison of recurrence rate and injection number

During follow-up, the recurrence rate of macular edema was 17.3% in the early group, lower than 26.5% in the intermediate group and 38.3% in the delayed group (P = 0.025). The mean total number of injections was 3.8 ± 1.2, 4.3 ± 1.4, and 4.9 ± 1.6 for the early, intermediate, and delayed groups, respectively, with significant differences among groups (*p* = 0.002) (**Table 4**).

**Table 4:** Comparison of recurrence rate and total injection number among groups.

Group	Recurrence	Injection number (times)
Early - stage group(n=67)	12 (17.9%)	3.76 ± 1.18 (n=67)
Mid - stage group(n=52)	14 (26.9%)	4.27 ± 1.39 (n=52)
Delayed group(n=29)	11 (37.9%)	4.93 ± 1.62 (n=29)
**F/χ²**	4.485	7.430
P	0.106	<0.001

### 3.5 Correlation between treatment delay and visual outcomes

Pearson correlation analysis revealed a significant negative correlation between treatment delay and six-month BCVA improvement (*r* = −0.454, *P* < 0.001), indicating that longer delays were associated with smaller visual gains.

### 3.6 Multivariable regression analysis of factors affecting visual outcomes in T2DM patients with BRVO

To identify independent factors affecting six-month BCVA improvement, multivariable linear regression included baseline clinical characteristics (age, diabetes duration, HbA1c, SBP, DBP, lipid levels), baseline ocular parameters (baseline BCVA, baseline CMT), treatment-related factors (injection number), lifestyle factors (sex, smoking history), and treatment delay. Outcome variables such as CMT change, recurrence, or 15-letter improvement were not included; only BCVA improvement was analyzed as the dependent variable.

The regression model (R² = 0.264, adjusted R² = 0.187) indicated that treatment delay was the only significant independent factor affecting six-month BCVA improvement (β = −0.008, 95% CI: −0.014 to −0.002, *P* = 0.010), suggesting that longer delays result in smaller visual gains. Other variables—including age, sex, diabetes duration, HbA1c, blood pressure, lipid levels, baseline BCVA, baseline CMT, injection number, smoking history, and group assignment were not statistically significant (*P* > 0.05) (**Table 5**). Collinearity diagnostics showed most variables had VIFs between 1–2, indicating low multicollinearity.

**Table 5:** Multivariable regression analysis of factors affecting visual outcomes in T2DM patients with BRVO.

Variables	β	95%CI	*P*	VIF
Treatment delay	-0.008	[-0.014, -0.002]	0.010	1.625
Age	-0.001	[-0.004, 0.003]	0.784	1.085
Diabetes duration	-0.001	[-0.005, 0.003]	0.663	1.053
HbA1c	-0.002	[-0.022, 0.018]	0.825	1.050
SBP	0.002	[-0.000, 0.003]	0.109	1.097
DBP	-0.001	[-0.004, 0.002]	0.517	1.177
Lipid	-0.027	[-0.065, 0.010]	0.147	1.049
Baseline BCVA	0.032	[-0.089, 0.152]	0.604	1.097
Baseline CMT	0.000	[-0.000, 0.000]	0.617	1.113
Injection number	0.001	[-0.015, 0.016]	0.928	1.158
Sex (Male)	-0.019	[-0.065, 0.027]	0.414	1.077
Smoking_history	0.033	[-0.011, 0.077]	0.144	1.045

### 3.7 ROC curve analysis

**Figure 3:**
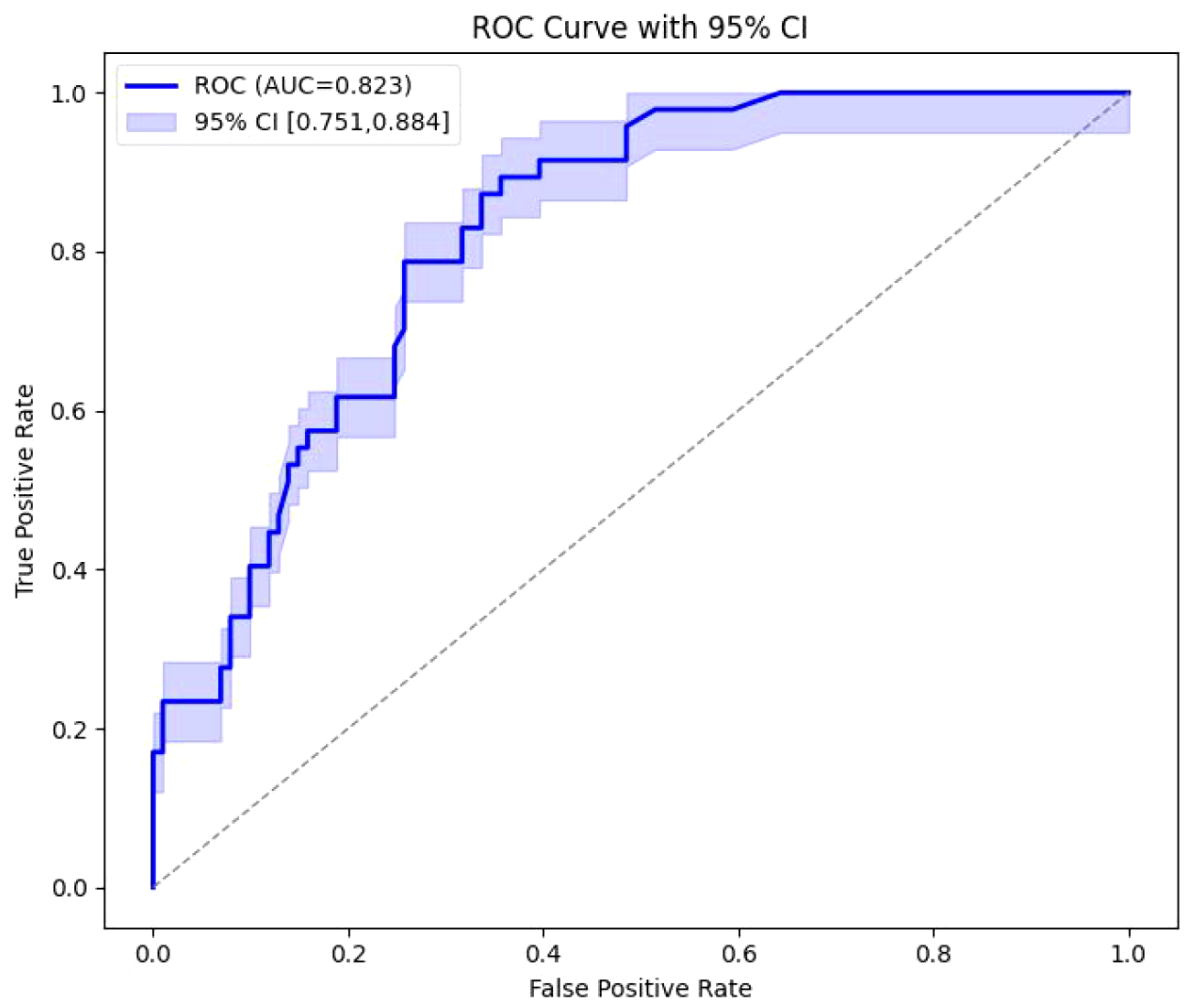
ROC curve analysis of delayed treatment for predicting insufficient 6-month visual improvement (ΔLogMAR <0.2).

ROC curve analysis showed that treatment delay had good predictive value for insufficient six-month visual improvement (ΔLogMAR <0.2), with an area under the curve (AUC) of 0.823 (95% CI: 0.751–0.884, *P* < 0.001). Using 14 days as the cutoff, sensitivity was 89.4% and specificity was 61.4% (**Figure 3**).

## 4 Discussion

BRVO-induced macular edema primarily results from retinal microcirculatory disturbances following venous occlusion, leading to endothelial dysfunction, increased vascular permeability, and elevated VEGF expression (14). Persistent macular edema can cause swelling of the retinal nerve fiber layer, photoreceptor damage, and disorganization of the ganglion cell layer, thereby limiting visual recovery (15). Delayed presentation prolongs the duration of edema, resulting in irreversible retinal structural changes such as photoreceptor outer segment degeneration and macular nerve fiber damage (16). Therefore, the timing of ranibizumab injection has a substantial impact on therapeutic outcomes. In real-world practice, the clinical condition at presentation is highly heterogeneous, and the benefit of treatment timing may vary with disease severity. In theory, early treatment in a very severe state may not fully reverse established structural damage, whereas a short delay in a mild case may still allow satisfactory recovery. We acknowledge this complexity. In the present study, however, this potential bias was partially mitigated by strict inclusion criteria and comparable baseline BCVA and CMT across groups, indicating a relatively homogeneous disease stage at enrollment. Within this clinically comparable window, treatment delay remained the only independent predictor of visual improvement. Therefore, our findings should be interpreted as applicable to patients presenting within a potentially reversible phase of BRVO-ME, rather than as a universal rule across all severity stages.

From a biological perspective, the apparent sensitivity of outcomes to a relatively short delay (7–14 days) may seem counterintuitive. However, “delay” in this context does not merely represent calendar time, but rather the duration of sustained macular edema and VEGF exposure. Experimental and clinical evidence suggests that retinal tissue may exhibit a biological threshold beyond which structural recovery becomes limited. Once this threshold is crossed, anti-VEGF therapy can reduce retinal thickness but cannot fully restore neurosensory architecture (11). Baseline BCVA and CMT reflect a static snapshot at presentation, whereas treatment timing determines whether the retina remains within a reversible window. Lifestyle and systemic factors may influence disease progression over longer time scales, but in the acute phase of BRVO-ME, the duration of untreated edema appears to be the dominant driver of irreversible damage.

Diabetic microangiopathy, endothelial dysfunction, and chronic low-grade inflammation may plausibly render the retina more vulnerable to prolonged edema and ischemia, thereby amplifying the biological consequences of delayed intervention in this population. However, because the present study did not include a non-diabetic BRVO cohort, this hypothesis cannot be directly tested. Our findings should therefore be interpreted as characterizing the timing effect within diabetic BRVO, rather than establishing a differential effect between diabetic and non-diabetic populations.

Ranibizumab is a recombinant humanized monoclonal antibody targeting VEGF-A that reduces macular edema by inhibiting VEGF-mediated vascular permeability and neovascularization (17). In this study, the early treatment group (≤7 days) demonstrated significantly greater BCVA improvement and the largest CMT reduction compared with the intermediate (8–14 days) and delayed (>14 days) groups, indicating that early intervention with ranibizumab more effectively blocks the VEGF pathway and improves the macular microenvironment. These findings are consistent with previous reports; for example, Brown et al. demonstrated that early anti-VEGF intervention in BRVO-ME patients significantly shortens the duration of macular edema and enhances visual improvement (18), which our results further validate in terms of patient grouping and outcome measures.

In our cohort of 148 T2DM patients with BRVO-ME, delayed presentation was the only independent factor affecting six-month BCVA improvement, with longer delays associated with smaller visual gains. This suggests that timing may play a more critical role in anti-VEGF therapy than other clinical characteristics.

Notably, injection number was not a significant factor in multivariable regression. A possible explanation is that the study adopted a “3+PRN” regimen: early intervention reduced recurrence and, therefore, injection number in the early group was relatively low, while in the delayed group, additional injections could not fully reverse structural damage already incurred, limiting BCVA improvement. This aligns with previous findings: Wecker et al. reported that while injection number influences short-term CMT reduction, long-term BCVA improvement is more affected by edema duration and baseline retinal damage (19).

Other variables, including age, sex, diabetes duration, HbA1c, blood pressure, and lipid levels, were not significant in multivariable analysis. This may relate to the relative homogeneity of our sample: all patients had T2DM with BRVO-ME, the age range was limited (mean ~65 years), and most had controlled blood glucose and blood pressure, potentially diminishing the observable effect of these variables on visual improvement. Additionally, baseline BCVA and CMT, although correlated with improvement in univariate analysis, may be overshadowed by the strong effect of treatment delay—a “delay-priority” effect. This partially aligns with previous studies, such as the SCORE study, which suggested that while initial edema severity correlates with visual improvement, its predictive value is reduced when early intervention is applied (20).

Our results are consistent with meta-analyses by Scott et al., which found that delayed anti-VEGF treatment is closely associated with poor visual outcomes (21). Unlike studies emphasizing baseline CMT or initial BCVA as primary predictors, our multivariable analysis suggests that in T2DM patients with BRVO-ME, the timing of intervention may be more critical than baseline structural indices. Furthermore, ROC analysis in this study indicated that using 14 days as a cutoff predicted insufficient six-month BCVA improvement with 89.4% sensitivity and 61.4% specificity, providing a clinically actionable reference window. This aligns with previously reported 7–10 day delay thresholds and further validates the delay threshold in clinical practice.

## 5 Clinical significance and limitations

This study emphasizes the importance of early clinical intervention: even in the presence of variability in baseline characteristics and lifestyle factors, delayed presentation remains the primary modifiable factor affecting BCVA improvement. Clinically, patient outcomes can be optimized by: (1) increasing awareness among diabetic patients for ophthalmic follow-up and prompting early evaluation for BRVO symptoms; (2) streamlining outpatient and emergency workflows to reduce the interval from diagnosis to treatment; (3) implementing rapid assessment and early intervention strategies for high-risk groups (e.g., elderly patients, those with hypertension or poor glycemic control). These findings also provide empirical support for treatment guidelines, suggesting that early ranibizumab intervention maximizes VEGF pathway blockade, minimizes macular structural damage, and enhances long-term visual recovery.

This study has several limitations. First, the single-center, retrospective design may limit external generalizability. Second, retrospective data are subject to selection and information bias, such as inaccuracies in presentation timing and OCT measurements. Third, potential confounders, including types of diabetic complications, socioeconomic status, and lifestyle factors, were not fully accounted for, which may influence BCVA outcomes. Fourth, although multivariable regression controlled for known variables, the relatively small sample size may limit statistical power. Finally, follow-up was limited to six months, leaving long-term visual recovery uncertain.

## 6 Conclusion

The results of this study indicate that, in patients with T2DM complicated by BRVO-ME, delayed presentation is an independent factor affecting six-month improvement in BCVA, whereas other baseline clinical characteristics and injection number have limited impact. Clinically, emphasis should be placed on early recognition and timely intervention to optimize visual outcomes. Given the single-center, retrospective design, these findings should be interpreted with caution. Future multicenter, prospective studies are warranted to validate these results and further investigate the mechanisms by which treatment delay affects long-term macular structure and visual function.

## Data Availability

The original contributions presented in the study are included in the article/supplementary material. Further inquiries can be directed to the corresponding author.
